# Disclosing mental health in medical education: A global qualitative meta‐synthesis

**DOI:** 10.1111/medu.70240

**Published:** 2026-05-08

**Authors:** Sameera Aljuwaiser, Gwyndaf Roberts

**Affiliations:** ^1^ School of Medicine, Faculty of Medical Sciences Newcastle University Newcastle Upon Tyne UK

## Abstract

**Introduction:**

Mental health disorders among medical students represent a persistent global challenge. Disclosure, defined as the act of revealing a mental health disorder to peers, educators or institutions, is shaped by cultural norms, institutional structures and professional expectations. Despite growing awareness of well‐being, disclosure remains complex and fraught with stigma, confidentiality concerns and fears of professional repercussions. This international qualitative meta‐synthesis explores how medical students experience and navigate mental health disclosure, aiming to clarify the social, cultural and institutional forces that shape disclosure decisions. It further seeks to inform policies that create psychologically safe learning environments.

**Methods:**

A systematic search of nine databases (updated December 2025) identified peer‐reviewed qualitative and mixed‐methods studies published between 2013 and 2025. Data were synthesised using Thomas and Harden's thematic synthesis method within an interpretivist paradigm. Reporting followed the enhancing transparency in reporting the synthesis of qualitative research (ENTREQ) and preferred reporting items for systematic reviews and meta‐analyses (PRISMA) guidelines, and methodological quality was assessed using the critical appraisal skills programme (CASP) checklist. Confidence in the review findings was evaluated using confidence in the evidence from reviews of qualitative research (GRADE‐CERQual). The review protocol was registered with the international prospective register of systematic reviews (PROSPERO; CRD42024521037).

**Results:**

Eighteen studies from seven countries (*n* = 379 participants) met the inclusion criteria. Six overarching themes and 16 subthemes were identified. Key barriers to disclosure included stigma, professional anxiety, confidentiality concerns, institutional rigidity and uncertainty about support services. Peer influence emerged as the only consistent facilitator of disclosure. The findings illustrate how medical culture, systemic structures and social expectations intersect to sustain nondisclosure and limit access to support.

**Conclusion:**

Disclosure of mental health disorders in medical education is a negotiated, relational process embedded within socio‐cultural and institutional power dynamics. This synthesis advances understanding by situating disclosure within disability and critical theory frameworks, highlighting how ableism and professional identity norms perpetuate silence. Addressing these structural forces through inclusive policies and psychologically safe learning cultures is essential to normalising openness and supporting the well‐being of future doctors.

## Abstract 2

New study reveals barriers like stigma and systemic obstacles to mental health disclosure among medical students. Calls for reforms to foster openness & resilience. #MentalHealth #MedEd

## 1 INTRODUCTION

Mental health disorders remain a significant and persistent challenge within medical education.^1^ The demanding nature of medical training, characterised by intensive academic expectations, prolonged clinical placements and frequent high‐stakes examinations, renders medical students particularly vulnerable to psychological distress and mental health disorders.^2^, ^3^, ^4^ Additional stressors exacerbate this risk, including financial debt, sleep deprivation and relationship pressures.^5^, ^6^, ^7^, ^8^ Consequently, medical students consistently report higher rates of mental health disorders compared to their age‐matched peers.^9^

Despite this high prevalence, many students face barriers to disclosing their mental health disorders.^10^ Disclosure, defined here as the act or process of revealing one's mental health disorder to another person or institution, whether formally (e.g. to occupational health or academic advisors) or informally (e.g. to peers or mentors), is a complex, ongoing process shaped by social, institutional and cultural contexts.^11^ It is not a single event but a strategic negotiation of identity and safety, influenced by perceptions of stigma, professional expectations and systemic ableism within medical education.^12^, ^13^

This review draws on conceptualisations of disclosure developed in disability studies, including Evans's framing of uncovering or covering as a strategic negotiation^14^ and Jain's distinction between logistical disclosure (to access adjustments) and political disclosure (to challenge structural norms).^15^ These perspectives inform the understanding of disclosure as relational, contextual and embedded within institutional power.^16^

Untreated or undisclosed mental health disorders can have detrimental consequences, including delayed treatment, reduced academic performance and attrition from medical programmes.^17^ Early disclosure and intervention are therefore crucial for enabling timely access to support, accommodations and treatment. However, disclosure also carries risks, such as fear of judgement, breaches of confidentiality and adverse career expectations.^18^ Where disclosure is safe and supported, timely adjustments can alleviate distress and aid academic progression.^19^

In the United Kingdom, the General Medical Council (GMC), the national regulatory body responsible for medical education and professional standards, mandates that medical schools provide adequate mental health support and reasonable adjustments for students with mental health disorders.^20^ Similar frameworks exist globally, such as the Liaison Committee on Medical Education (LCME) standards in North America^21^ and the World Federation for Medical Education (WFME) global standards.^22^ Although these regulatory expectations aim to ensure inclusion, evidence suggests that barriers to disclosure persist internationally.^23^ Prior international research consistently shows low disclosure rates among medical students despite rising prevalence and expanding institutional well‐being policies, indicating that barriers remain entrenched.^24^, ^25^

Existing research has primarily examined prevalence, stigma and attitudes towards mental health within medical education, but relatively few studies have explored the lived experiences and decision‐making processes surrounding disclosure. Previous reviews have seldom integrated findings across national contexts or applied a disability studies lens that recognises ableism, sanism and institutional culture as structuring forces in disclosure.

This qualitative meta‐synthesis aims to address this gap by synthesising international evidence on medical students' experiences of disclosing mental health disorders. It seeks to

1. identify factors influencing students' decisions to disclose or conceal mental health disorders;
2. explore how institutional, cultural and professional norms shape disclosure processes; and
3. inform policies and practices that promote psychologically safe and inclusive learning environments.

## 2 METHODS

### 2.1 Study design

This study adopted a qualitative meta‐synthesis approach to integrate and interpret qualitative evidence on medical students' experiences of mental health disclosure.^26^ The review followed Lachal et al.'s six‐step framework^26^ and ENTREQ reporting standards.^27^ The protocol was registered with PROSPERO (CRD42024521037) and reported in line with preferred reporting items for systematic reviews and meta‐analyses (PRISMA) guidelines.^28^ ENTREQ and PRISMA checklists are openly available at https://doi.org/10.25405/data.ncl.29223848.

Qualitative meta‐synthesis was chosen because the review question addresses students' experiences and decision‐making processes, which are best explored through inductive integration of qualitative findings. Thematic synthesis enables the translation of concepts across studies to generate higher‐order themes that transcend individual contexts. This approach, grounded in a constructivist epistemology and interpretivist paradigm, supports the development of an explanatory model of disclosure decisions across diverse settings, recognising that meaning is co‐constructed between researchers and participants.

Reflexivity was integral: the lead researcher (a clinician) maintained a reflexive journal to document assumptions, disciplinary influences and interpretive decisions. The lead researcher's clinical background and the co‐author's expertise in medical education informed interpretation, with reflexive awareness of how professional identity and disciplinary perspective may have influenced analytic framing. This stance acknowledges that thematic synthesis involves interpretive engagement rather than passive aggregation of data.

### 2.2 Search strategy

A systematic search of nine databases, Medline, Embase, PsycINFO, CINAHL, British Education Index, Education Database, ERIC, Scopus and Web of Science, was conducted covering the period from 2013 to 2025 and updated on 17 December 2025. The 2013 cut‐off aligns with a global policy shift towards prioritising mental health in education, marked by the launch of the WHO's Mental Health Action Plan 2013–2020.^29^

Search terms were structured using the sample, phenomenon of interest, design, evaluation, and research type (SPIDER) tool to capture qualitative studies exploring disclosure experiences among medical students.^30^ Forward citation tracking and manual reference checks supplemented database searches. Full search strategies are openly available at https://doi.org/10.25405/data.ncl.29223848.

To capture a comprehensive range of experiences, the National Institute for Health and Care Excellence (NICE) definition of ‘common mental health problems’ was expanded to include mental health disorders such as anxiety disorders, bipolar disorder, burnout, depression, eating disorders, obsessive‐compulsive disorder, post‐traumatic stress disorder, psychological distress, panic disorder, social anxiety disorder, stress and suicidal ideation.^31^

Grey literature was excluded to maintain methodological rigour, consistency in peer‐review standards and reproducibility of findings. Key institutional reports (e.g. GMC and LCME) were reviewed narratively to contextualise results.

### 2.3 Eligibility criteria

Eligible studies were qualitative or mixed‐methods research (with extractable qualitative data) involving medical students' experiences or perspectives on mental health disclosure. Excluded were quantitative studies, editorials, conference abstracts and studies without primary qualitative data. Only English‐language publications from 2013–2025 were included. The inclusion criteria, structured via the SPIDER framework,^30^ are openly available at https://doi.org/10.25405/data.ncl.29223848.

### 2.4 Screening and study selection

Screening followed PRISMA guidelines.^28^ Duplicates were removed, and two reviewers (S. A. and G. R.) independently screened titles, abstracts and full‐texts using Rayyan™ and Endnote™. Disagreements were resolved through discussion. Reasons for exclusion are openly available at https://doi.org/10.25405/data.ncl.29223848.

The critical appraisal skills programme (CASP) qualitative checklist was used to assess the methodological and reporting quality of each included study. CASP was applied as a domain‐based appraisal tool, and studies were categorised as having minor, moderate or major methodological concerns based on CASP domains. No studies were excluded on quality grounds, recognising that poor reporting does not necessarily imply invalid results. However, studies with major concerns were treated with caution during interpretation and contributed less weight in developing analytical themes. These quality appraisals informed the GRADE‐CERQual assessments, specifically within the ‘methodological limitations’ domain for each synthesis finding.

### 2.5 Data extraction and analysis

Data were extracted into a customised Microsoft Excel® sheet, capturing study design, context, participant characteristics and key findings. Missing data were requested from study authors. An example extraction sheet is openly available at https://doi.org/10.25405/data.ncl.29223848.

Thematic analysis followed Thomas and Harden's three‐stage thematic synthesis method.^32^ Relevant text, primarily from the results and discussion sections, was coded line by line and organised into descriptive themes to capture recurring patterns across studies. Analytical themes were then developed through interpretive synthesis and researcher dialogue, providing deeper conceptual insights into medical students' experiences of mental health disclosure. NVivo© software supported coding, theme development and audit trail management.

Preliminary coding identified 29 themes, which were synthesised into six principal themes and 16 subthemes representing barriers and facilitators to disclosure. Analytical themes were iteratively refined to ensure coherence and alignment with the dataset. The GRADE‐CERQual approach evaluated confidence in each finding based on methodological quality, coherence, adequacy and relevance. Findings were translated into evidence statements to support their credibility.^33^

## 3 RESULTS

### 3.1 Study selection

A systematic search conducted on 20 March 2024 and updated on 17 December 2025 identified 3666 records. After removing duplicates, 2819 titles or abstracts were screened, and 2513 were excluded as irrelevant, leaving 306 for full‐text review. Eighteen studies met inclusion criteria.^34^, ^35^, ^36^, ^37^, ^38^, ^39^, ^40^, ^41^, ^42^, ^43^, ^44^, ^45^, ^46^, ^47^, ^48^, ^49^, ^50^, ^51^ The PRISMA flow diagram is presented in Figure 1.

**FIGURE 1:**
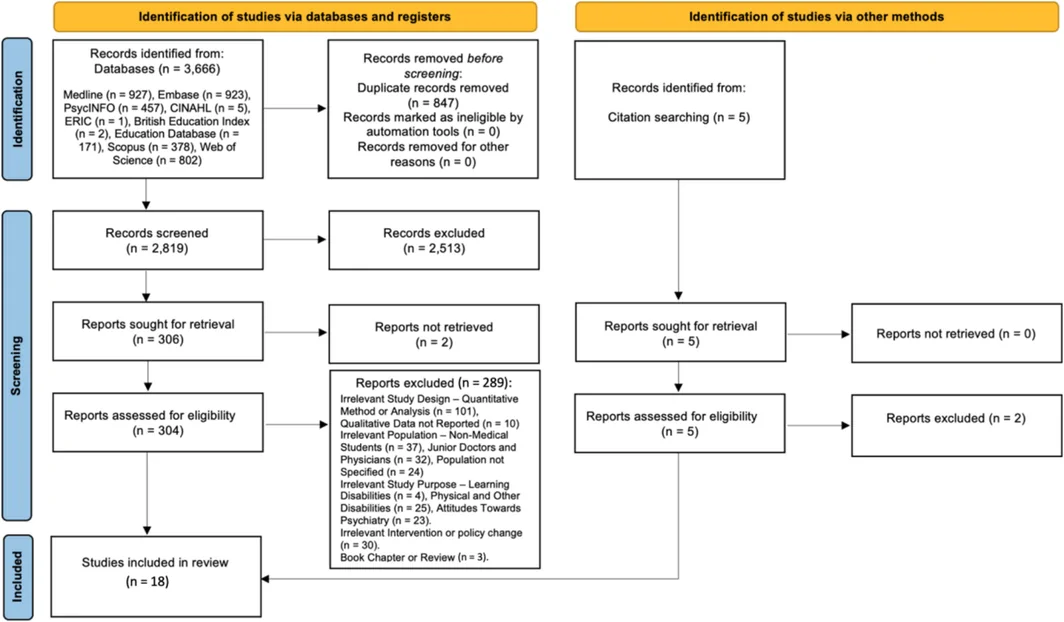
Study flow diagram according to the preferred reporting items for systematic reviews and meta‐analysis (PRISMA) guidelines. [Color figure can be viewed at wileyonlinelibrary.com]

### 3.2 Characteristics of included studies

The 18 included studies involved 379 medical students and were published between 2017 and 2025. Eleven were based in the United Kingdom, two in Canada and one each in Australia, Hungary, the Philippines and the United States; one study compared UK and Singapore cohorts.

Twelve studies focused on undergraduate students, three included both undergraduates and postgraduate learners, and three focused solely on postgraduate students. Four studies were mixed methods (qualitative data extracted only).

Theoretical frameworks included Constructivist Grounded Theory (*n* = 2), Pragmatism (*n* = 1) and Constructivism (*n* = 1); 14 used generic qualitative designs without specified frameworks. Data were collected via interviews, questionnaires and focus groups and analysed using thematic analysis (*n* = 10), grounded theory (*n* = 1), descriptive phenomenology (*n* = 1), content analysis (*n* = 3), framework analysis (*n* = 2) and unspecified thematic analysis (*n* = 1).

Three studies addressed the COVID‐19 pandemic. One study focused on psychological distress, another on occupational stress and the remaining 16 on various mental health disorders. Thirteen studies did not require participants to have a clinical diagnosis, whereas five focused specifically on students with self‐disclosed or formally diagnosed mental health disorders. The characteristics of included studies are openly available at https://doi.org/10.25405/data.ncl.29223848.

### 3.3 Methodological limitations

Seventeen studies clearly stated aims, and all described methodologies. Only one study^39^ adequately reported the researcher–participant relationship and its potential influence on findings. All studies detailed their data analysis processes, presented results transparently and highlighted contributions to existing knowledge. Ethical considerations were reported across all studies, although five did not report Research Ethics Committee approval identifiers or numbers.^34^, ^39^, ^47^, ^49^, ^50^ CASP quality appraisal summaries are openly available at https://doi.org/10.25405/data.ncl.29223848.

### 3.4 Overview of thematic findings

Thematic analysis identified six principal themes. Five themes, comprising 14 subthemes, captured barriers to disclosure, whereas one theme, comprising two subthemes, captured facilitators. Thematic structure, outlining specific barriers and facilitators across the included studies, is openly available at https://doi.org/10.25405/data.ncl.29223848. These themes were subsequently synthesised to explore their interrelationships and the broader contextual dynamics shaping disclosure decisions.

### 3.5 Theme interrelationships

Across studies, six overarching themes shaped medical students' decisions to disclose or conceal mental health disorders: Theme 1: social conformity, Theme 2: professional anxiety, Theme 3: medical education environment, Theme 4: systemic and institutional obstacles, Theme 5: ambiguities and Theme 6: peer influence (facilitator).

These themes were interconnected rather than discrete, reflecting the complex, multi‐layered nature of disclosure decisions (Figure 2). Theme 1: social conformity, driven by societal stigma and cultural expectations, fed into Theme 2: professional anxiety surrounding competence, fitness‐to‐practise and career progression. Theme 3: education environment, characterised by competition, resilience norms and confidentiality concerns, amplified these pressures by normalising silence and performance ideals. Theme 4: systemic and institutional obstacles, such as rigid administrative policies and scheduling constraints, further limited access to timely support, whereas Theme 5: ambiguities, including uncertainty about services and symptom recognition, delayed help‐seeking until crises emerged. By contrast, Theme 6: peer influence functioned as the only consistent facilitator, moderating the effects of Themes 1–5 by normalising help‐seeking and modelling safe disclosure behaviours.

**FIGURE 2:**
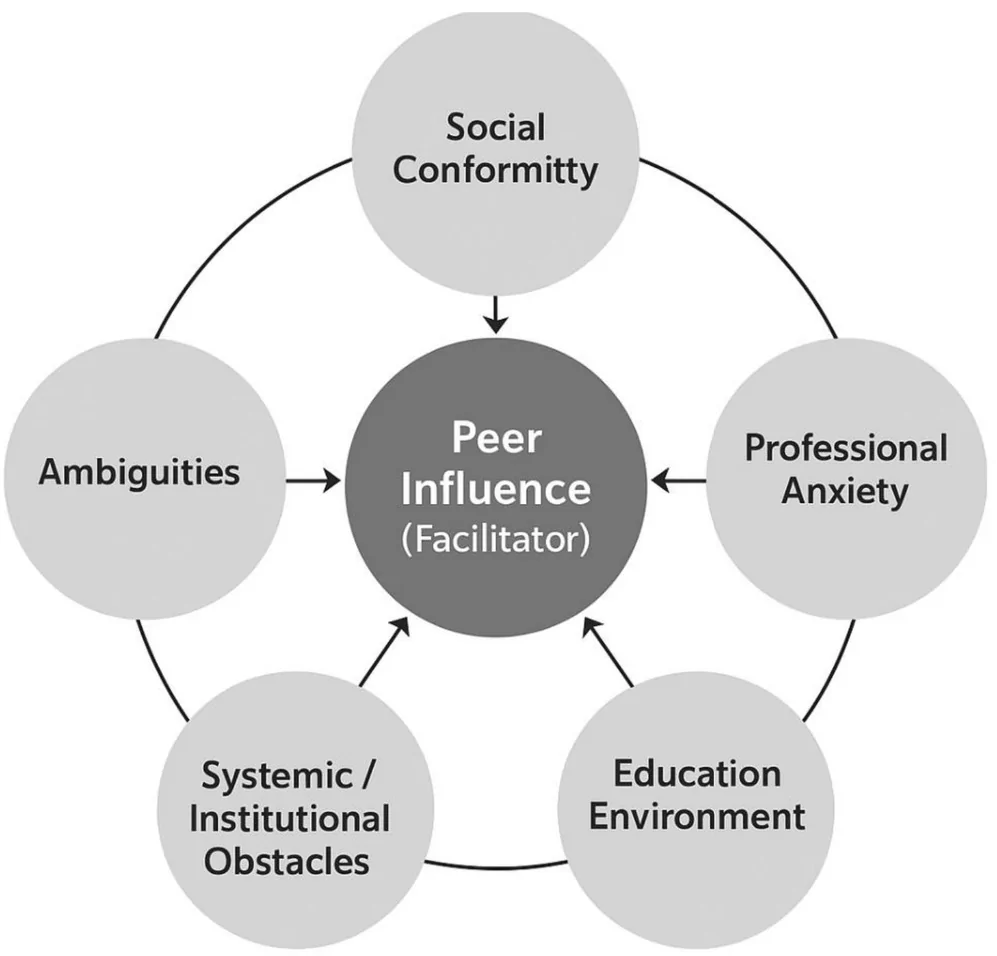
Theme map of interrelationships among Themes 1–6.

For definitional clarity, references to ‘culture’ in this synthesis denote (a) societal culture, referring to norms and beliefs within the broader community; and (b) the culture of medicine or medical school, encompassing professional socialisation, performance norms and the hidden curriculum. These terms are used explicitly throughout to maintain conceptual precision.

Institutional stressors refer to experiential pressures within programmes (e.g. competition and resilience expectations) captured under Theme 3, and systemic or institutional obstacles refer to structural barriers created by policies and processes (e.g. attendance rules, leave procedures and scheduling or placement logistics) captured under Theme 4. A matrix showing which studies supported each theme is openly available at https://doi.org/10.25405/data.ncl.29223848.

### 3.6 Confidence in synthesis findings (GRADE‐CERQual)

Confidence in each review finding was assessed using GRADE‐CERQual. CERQual evaluates confidence in qualitative synthesis findings based on four domains: methodological limitations of the contributing studies, coherence of findings, adequacy of supporting data and relevance to the review question. These are summarised in a Summary of Qualitative Findings table, with detailed Evidence Profiles openly available at https://doi.org/10.25405/data.ncl.29223848.

Findings supported by rich, consistent data from multiple high‐quality studies were rated with higher confidence. Where contributing studies had methodological concerns (assessed using CASP), or where data were limited, incoherent or less relevant, confidence ratings were downgraded.

### 3.7 Review findings

This meta‐synthesis identifies six themes influencing medical students' mental health disclosure: social conformity, professional anxiety, the medical education environment, systemic obstacles, ambiguities in addressing mental health and peer influence. Stigma, career fears and institutional pressures create significant barriers, whereas peer support creates solidarity and normalises disclosure.

#### 3.7.1 Theme 1: Social conformity

This theme explores social conformity, where societal norms, stigma and broader cultural expectations shape students' willingness to disclose mental health disorders.

##### Stigma as a barrier

Stigma emerged as a major barrier, as detailed in 11 of the included studies, with students internalising beliefs such as ‘*Doctors don't get sick*’^50^ and fearing disclosure signals incompetence. Misconceptions like ‘*The GMC has a rule that doctors have to be healthy*’^50^ reinforced this fear.

Internalised stigma contributed to self‐doubt and delayed help‐seeking, with one student describing it as ‘*an internalised fear*’.^37^ Although COVID‐19 temporarily reduced stigma, concerns persisted about returning to prior attitudes.^42^ Insensitive institutional responses, like ‘*Oh, you should know better*’,^43^ further discouraged disclosure.

##### Societal cultural beliefs as a barrier

Five studies showed that societal cultural norms (broader non‐medical cultural norms) often framed mental health as taboo. Students reported views of mental health as ‘*a myth*’^38^ or ‘*all in your head*’.^41^ In cultures emphasising endurance, students were expected to endure struggles silently: ‘*You are meant to endure things*’.^47^ Others described mental health as ‘*not something that is spoken about*’,^45^ reinforcing secrecy.

##### Fear of reputational damage

Seven studies found that students feared being seen as ‘*not tough enough for medicine*’^34^ or ‘*a failure*’.^50^ Concerns about permanent records and academic setbacks led some to describe disclosure as ‘*the end of the world*’^39^ driving concealment and discreet help‐seeking.^35^

#### 3.7.2 Theme 2: Professional anxiety

This theme explores balancing career demands with well‐being, as medical students with mental health disorders navigate fears of incompetence, expulsion and long‐term consequences.

##### Fear of fitness to practise procedures

Eight studies showed that students feared fitness to practise investigations could lead to expulsion or harm career prospects: ‘*I can understand why people would want to hide fitness to practice issues*’.^50^ Concerns about fairness and unclear procedures heightened anxiety, with one student stating: ‘*I still don't know whether I'm going to be able to work in August*’.^43^

##### Career repercussions

Fears about long‐term career impacts deterred disclosure, as detailed in five studies. Students worried about being ‘*seen as a liability*’ during speciality training applications.^51^ Others questioned: ‘*What if it … affects my medical career later?*’.^39^ Some avoided disclosure entirely, admitting, ‘*I didn't put that [mental health disorder] down*’.^48^

#### 3.7.3 Theme 3: The medical education environment

This theme examines how institutional pressures, including culture, competition, high expectations and lack of trust, affect medical students' willingness to disclose mental health disorders.

##### Confidentiality concerns as barriers

Nine studies found that students expressed fears about privacy, worrying disclosures might not remain confidential. Concerns included ‘*Even the confidentiality of the GP visit can be in doubt*’^50^ and ‘*I'm potentially saying [something] to a future colleague*’.^46^ Many distrusted institutional support, fearing disclosures could lead to scrutiny or penalties. One student stated: ‘*If I am telling her [clinical advisor] something, I am telling the school*’.^45^ Others worried universities might ‘*look for evidence to chuck you out*’.^34^ Institutional support mechanisms were sometimes viewed as surveillance rather than help: ‘*It was another way of putting me back on their radar*’.^49^

##### Competition as a barrier

The competitive culture in medical schools added pressure to maintain appearances of competence, discouraging disclosure, as seen in eight studies. Students described their peers as ‘*highly self‐critical, highly ambitious*’^50^ and felt isolated among *‘over‐achievers*’.^43^ Competition intensified fears of being perceived as weak: ‘*People don't like to admit that they've got problems*’.^50^ Imposter syndrome further exacerbated anxiety, with one student describing feeling that others were ‘*so much smarter and better*’.^43^

##### Resilience expectations as barriers

Eight studies found that medical schools often promoted emotional toughness, discouraging disclosure. Students felt pressured to appear ‘*smart … able to cope with pressure*’.^39^ Many internalised stress, normalising symptoms like ‘*nervousness, chest pain … and abdominal discomfort*’.^49^ Emotional detachment was viewed as a professional necessity, reinforced by observing senior colleagues maintain composure during difficult situations.^50^ Students admitted ‘*keeping everything hidden*’^43^ and managing struggles privately to avoid appearing weak.^38^

##### Cultural norms and institutional stressors

Eleven studies reported that medical school culture often normalised stress, with students describing it as ‘*stressful*’^34^ and feeling ‘*drained*’.^48^ Mental health struggles were sidelined in favour of academic performance: ‘*Focus on your studies, not yourself*’.^34^ Rotations and placements added to feelings of isolation and exhaustion: ‘*You move to different cities almost every 2 weeks*’.^51^ A perceived double standard also emerged, with empathy promoted for patients but not extended to students: ‘*We talk about illnesses as if no one in the room could possibly have it*’.^37^

#### 3.7.4 Theme 4: Systemic and institutional obstacles

This theme emphasises systemic barriers to mental health disclosure, including course structure and logistical challenges.

##### Administrative barriers

Five studies outlined that rigid policies, bureaucratic hurdles and unclear procedures make accessing mental health support difficult. Strict attendance rules prioritise presence over well‐being: ‘*[The medical school] are very quick on pointing out … when you haven't turned up, but they are not very quick to react when you need that help*’.^50^ Sick leave processes are burdensome, requiring extensive documentation and planning: ‘*I didn't take … leave because I didn't really understand how to*’.^51^ Policies lack flexibility, forcing students to attend classes despite poor health: ‘*Even if you're not well, you have to keep going*’.^51^

##### Geographical and time constraints

Eight studies revealed that demanding schedules and clinical rotations limit students' ability to seek timely support. Appointments often clash with commitments: ‘*I just didn't have the time … didn't know when to fit that in*’.^49^ Frequent relocations disrupt access to care and support networks: ‘*Not having easy access to any of your primary care people*’.^51^

##### Curriculum stressors

Seven studies reported that emotionally taxing content and unpreparedness for critical incidents increase distress. Clinical experiences can be overwhelming: ‘*Watching someone die or trying to save someone's life*’.^48^ Academic pressures often overshadow mental health concerns, especially during exams: ‘*Mental health … wasn't the priority*’.^49^ A relentless workload creates a sense of overwhelm: ‘*You don't know where to stop*’.^39^

#### 3.7.5 Theme 5: Ambiguities in addressing mental health

This theme highlights challenges in recognising and addressing mental health disorders due to limited awareness of support services and symptoms.

##### Uncertainty about support services

Eight studies found that many students are unaware of available support, hindering help‐seeking: ‘*I wasn't aware of anyone available*’.^49^ Others find services ineffective or hard to access: ‘*I had to do a lot of chasing to find the right person*’.^45^ Doubts about service quality further deter disclosure: ‘*They are not adequate at all*’.^38^

##### Failure to recognise symptoms

Six studies revealed that students often struggle to identify symptoms or determine when help is needed: ‘*I wouldn't know when I should go to a psychologist*’.^38^ Help is often sought only when symptoms become severe: ‘*I couldn't even leave the flat without having a panic attack*’.^43^ A lack of insight and understanding about mental health disorders creates further delays: ‘*I never realised that experience is totally different from reading about it*’.^49^

#### 3.7.6 Theme 6: Peer influence

This theme reveals the positive impact of peer support, where supportive relationships foster a safe environment for sharing struggles.

##### Peers' positive experiences

Three studies reported that hearing about peers' positive experiences with mental health resources encourages disclosure: ‘*It's really good … They really do want to help*’.^36^ Encounters with empathetic professionals reinforce this support: ‘*Just having her listen in itself was very healing*’.^49^ These shared experiences offer a sense of reassurance, which, by contrast with the more prominent barriers, helps normalise disclosure.

##### Peer support networks

In six studies, conversations with peers normalise struggles and reduce isolation: ‘*Speaking to other people who are also stressed and struggling … I didn't feel like it was just me*’.^36^ Peer disclosures create solidarity and encourage help‐seeking: ‘*A girl who shared first … then I … was like, “I actually have (it) myself”*’.^45^ This process of shared disclosure creates a safe, supportive space that actively challenges the stigma surrounding help‐seeking, offering a counterbalance to the prominent barriers.

## 4 DISCUSSION

This qualitative meta‐synthesis provides a conceptual synthesis of international evidence on medical students' experiences of disclosing mental health disorders. It highlights the complex interplay between socio‐cultural expectations, professional identity formation and systemic structures within medical education. The six themes illustrate how stigma, institutional culture and professional anxiety combine to discourage disclosure, whereas peer relationships function as the primary facilitator. These findings extend prior research by reframing disclosure not only as an individual decision but as a relational, contextual and political process situated within systems shaped by ableism and sanism.

### 4.1 Interpretation of findings

#### 4.1.1 Disclosure as a negotiated and relational process

The findings confirm that disclosure among medical students is rarely a single act; rather, it is an ongoing and negotiated process shaped by anticipated social, professional and institutional responses. Students weigh potential benefits, such as access to care, accommodations or emotional relief, against potential harms, including loss of confidentiality, professional stigma and jeopardised career prospects. This mirrors disability studies literature, which conceptualises disclosure as a strategic practice of ‘covering and uncovering’ within hierarchical institutions.^14^, ^15^

The persistence of fear surrounding disclosure, despite increasing institutional rhetoric on well‐being, emphasises a disconnect between formal policy and lived experience. Notably, major regulators and accreditors emphasise confidential access to support and separation of healthcare from academic assessment. The GMC advises that treatment should be managed separately from the medical school and that confidentiality policies should be clear and accessible to students.^20^ Similarly, LCME standards require that student health providers (including psychiatric or psychological counsellors) have no role in academic assessment or promotion and that student health records are securely protected for privacy and confidentiality.^21^ WFME standards specify that student counselling and support services should be accessible and confidential.^22^ Yet across studies, students remained doubtful that these safeguards function in practice, reporting fears of confidentiality breaches, documentation on academic records and career repercussions.^10^ Consequently, disclosure becomes a form of calculated risk‐taking, especially in cultures where the hidden curriculum rewards stoic perfectionism and frames vulnerability as incompatible with clinical credibility.^52^

#### 4.1.2 Stigma, ableism and the culture of medicine

Consistent with previous literature, stigma emerged as a central barrier.^53^ The medical ‘hidden curriculum’ reinforces a professional ideal of emotional invulnerability and relentless competence, manifestations of ableist and sanist norms that implicitly frame mental health disorders or disability as a deviation from the ‘ideal doctor’. These expectations marginalise students who experience psychological distress, positioning them as ‘at risk’ or ‘less professional’.^54^ In line with disability studies scholarship, these norms are embedded in professional identity formation and can render nondisclosure a practical survival strategy within hierarchical training environments.^14^, ^15^

In this context, the problem lies not in students' personalities but in medical culture and institutional arrangements. Jain's concept of the ‘capability imperative’ illustrates how medical training valorises a ‘selfless superhuman’ and upholds a cultural logic of compulsory hyper‐ablebodiedness and mindedness, rendering mental health disorders and disability legible as potential threats to perceived competence.^55^ Read alongside Jain's theorisation of legibility, disclosure can be understood as relational and political ‘legibility work’, in which students must make their lived experiences recognisable and legitimate within institutional systems that simultaneously evaluate, categorise and sanction.^56^ Under conditions of ableism, increased legibility may enable access to support and also heightening vulnerability to surveillance, stigma and exclusion.^14^, ^55^, ^56^ This helps explain why nondisclosure, across studies in this meta‐synthesis, repeatedly appeared not as individual reluctance but as a rational risk‐management strategy, particularly in contexts where students anticipated bias, breaches of confidentiality or misinterpretation of their performance.^57^, ^58^, ^59^

The patterns demonstrate that disclosure barriers extend beyond individual attitudes and are structurally produced through assessment regimes, cultural hierarchies and policy gaps that reinforce silence. Addressing these barriers, therefore, requires shifting away from individual‐level interventions (e.g. resilience training) towards structural reform of institutional practices and cultures that make disclosure safer, more credible and less burdensome.

#### 4.1.3 Systemic and institutional barriers

Students frequently described administrative and procedural barriers, rigid attendance rules, opaque fitness‐to‐practise procedures and insufficiently confidential support pathways.^60^ These findings echo international studies showing that institutional bureaucracy, rather than personal reluctance alone, sustains nondisclosure.^61^ The absence of formal systemic facilitators is striking, as across 18 studies, peer influence was the only consistent enabler of disclosure. Read through legibility, peers appear to act as informal ‘recognition infrastructures’, enabling students to test disclosure, receive validation and appraise institutional risk before becoming institutionally legible.^56^

Accordingly, institutions should prioritise structural enablers, such as streamlined mental health pathways, clear confidentiality protections and integrated well‐being offices independent of assessment bodies. Evidence from inclusive education research suggests that structural safeguards, such as separating academic and health records and introducing trusted intermediary roles, can facilitate disclosure and improve outcomes by reducing perceived risk and disclosure burden.^15^, ^19^

#### 4.1.4 Ambiguities and intersectional dimensions

Students' uncertainty about what constitutes a mental health disorder or when to seek help highlights enduring ambiguities within professional discourse. These ambiguities intersect with gender, cultural background, socio‐economic status and race, influencing perceptions of vulnerability and the acceptability of disclosure.^62^ For instance, learners from collectivist cultures may perceive disclosure as socially inappropriate, whereas those from minoritised backgrounds may fear compounding existing marginalisation.^63^ However, intersectional perspectives were largely absent from the primary studies, limiting the ability to explore these dynamics in depth. Future research should treat intersectionality as a core analytical lens to understand how ethnicity, gender, socio‐economic status and cultural context interact with disability‐related stigma in shaping disclosure experiences.^64^

### 4.2 Contribution to the field

This qualitative meta‐synthesis advances understanding of medical student mental health disclosure in three ways. First, it extends theorisation of disclosure as a social, relational and contextual process by mapping the findings onto Jain's constructs of legibility and the capability imperative, where disclosure emerges as legibility work undertaken within a training culture that valorises hyper‐capability and treats vulnerability as professionally risky.^56^ Second, it identifies a structural gap in current learning environments by showing that peer relationships were the only consistent facilitator across studies, suggesting that disclosure safety is currently sustained through informal networks rather than reliable institutional infrastructures. Third, it clarifies a policy‐practice paradox: even where regulators and accreditors require confidential support and separation from assessment, students continue to anticipate confidentiality breaches, documentation and career harm, indicating that institutional trust and power asymmetries, rather than awareness alone, remain central targets for reform.

### 4.3 Strengths

This meta‐synthesis represents the first comprehensive qualitative integration of medical students' perspectives on mental health disclosure. The review adhered to systematic review standards,^28^ including predefined eligibility criteria, transparent search strategies and robust appraisal methods, following ENTREQ, PRISMA and GRADE‐CERQual frameworks to ensure analytical depth and reproducibility. The search was initially conducted in March 2024 and updated in December 2025 to include the most up‐to‐date evidence. The inclusion of studies from multiple regions and methodological traditions enhances the richness and transferability of the findings. The convergence of results across diverse contexts strengthens confidence in the conceptual relevance and applicability of the emergent themes.

### 4.4 Limitations

The predominance of studies from high‐income, Anglophone contexts may limit global generalisability. However, research indicates that healthcare students globally experience similar mental health challenges, affirming the broader relevance of these insights.^9^, ^65^, ^66^, ^67^ The absence of reflexivity statements in several primary studies may have constrained interpretive depth, and reliance on self‐reported data introduces potential recall and social‐desirability bias. The limited representation of minority and marginalised groups also restricts conclusions regarding intersectional experiences. Despite these constraints, the consistency of findings across distinct cultural and educational settings enhances confidence in their conceptual robustness and wider applicability.

### 4.5 Implications and future directions

#### 4.5.1 Educational policy and institutional practice

Medical schools should implement clear, confidential disclosure protocols distinct from academic evaluation processes. Integrating occupational health or well‐being services independent of assessment bodies can protect privacy and build trust. Curricular initiatives should embed mental health literacy, self‐care and peer‐support training to normalise disclosure and early help‐seeking.

#### 4.5.2 Structural and cultural change

Addressing disclosure barriers requires confronting the underlying **ableism and perfectionism** that shape medical identity formation. Evidence from organisational psychology shows that cultures valuing vulnerability and authenticity improve psychological safety and reduce burnout.^68^ Embedding such values in medical education would reframe mental health as integral to professional competence rather than a deviation from it.

#### 4.5.3 Research directions

Future work should employ longitudinal designs to explore how disclosure attitudes evolve across different stages of medical training and during the transition into professional practice. It should also adopt intersectional and cross‐cultural approaches to capture the diversity of disclosure experiences across gender, ethnicity, socio‐economic status and cultural contexts. Evaluating interventions that modify institutional policy, such as anonymised reporting systems, confidential mentorship frameworks and independent well‐being services, will be crucial to understanding what creates safer disclosure environments. Extending this line of inquiry to other health professions, including nursing, dentistry and allied health disciplines, will help distinguish discipline‐specific challenges from barriers that are systemic across healthcare education.

## 5 CONCLUSION

Disclosure of mental health disorders among medical students remains a complex, high‐stakes process shaped by stigma, institutional structures and professional norms. This meta‐synthesis demonstrates that existing frameworks insufficiently address the relational and systemic nature of disclosure. Meaningful progress requires shifting from individual resilience models to institutional responsibility, cultivating environments where openness is protected, vulnerability is normalised and support is embedded in the fabric of professional education. By situating disclosure within disability and critical theory perspectives, this work extends understanding beyond prevalence or attitude studies, offering a foundation for structural reform and inclusive practice across global medical education systems.

## AUTHOR CONTRIBUTIONS

**Sameera Aljuwaiser:** Conceptualization; investigation; writing—original draft; methodology; formal analysis; data curation; validation; writing—review and editing. **Gwyndaf Roberts:** Methodology; supervision; writing—review and editing; validation; data curation; project administration.

## CONFLICT OF INTEREST STATEMENT

The authors declare no conflicts of interest.

## ETHICS STATEMENT

This article is a literature review and does not involve primary data collection. As such, ethical approval was not required.

## Data Availability

For the purpose of open access, the authors have applied a Creative Commons Attribution (CC BY) licence to any Author Accepted Manuscript version arising from this submission. All data created during this research will be openly available at https://doi.org/10.25405/data.ncl.29223848.
